# Plasma oxytocin in dry dairy cows after using a mechanical brush

**DOI:** 10.3168/jdsc.2025-0747

**Published:** 2025-05-12

**Authors:** Lena Skånberg, Sigrid Agenäs, Rupert Bruckmaier, Daiana de Oliveira, Linda Keeling

**Affiliations:** 1 Department of Applied Animal Science and Welfare, Swedish University of Agricultural Sciences, 750 07 Uppsala, Sweden; 2 Veterinary Physiology, Vetsuisse Faculty, University of Bern, CH-3001 Bern, Switzerland

## Abstract

Mechanical brushes are a common feature in loose housing systems for dairy cows and are suggested to be linked to positive welfare, yet the physiological effects of brush use remain unexplored. This study examined the influence of cows' mechanical brush use on plasma oxytocin (OT) levels and explored its relationship with which body regions were brushed by the cow. We predicted that this self-grooming would lead to an OT increase, suggesting calming effects and pleasant feelings from brush use similar to those found from gentle tactile stimulation in other mammals. Additionally, we expected an influence of the body region brushed, given previously documented heart rate and behavioral differences from allogrooming different cow body regions. Twelve dry (nonlactating) cows were observed, with 3 blood samples taken before and after brushing, during which each cow had sole access to a mechanical brush. Results showed that OT levels in the sample taken within the 2 min following brush use were higher than baseline value, but only in younger cows with ≤2 previous lactation periods. The increase was small but may be of biological relevance. Additionally, OT changes varied by the brushed body region, highlighting the importance of this factor in future studies investigating cows' brush use in relation to positive welfare.

## Highlights

• OT levels were measured in 12 dairy cows before and after brush use.
• OT levels increased among younger cows within the first 2 minutes after brushing.
• The duration of brushing different body regions influenced the OT increase.

## Graphical Abstract

**Figure:**
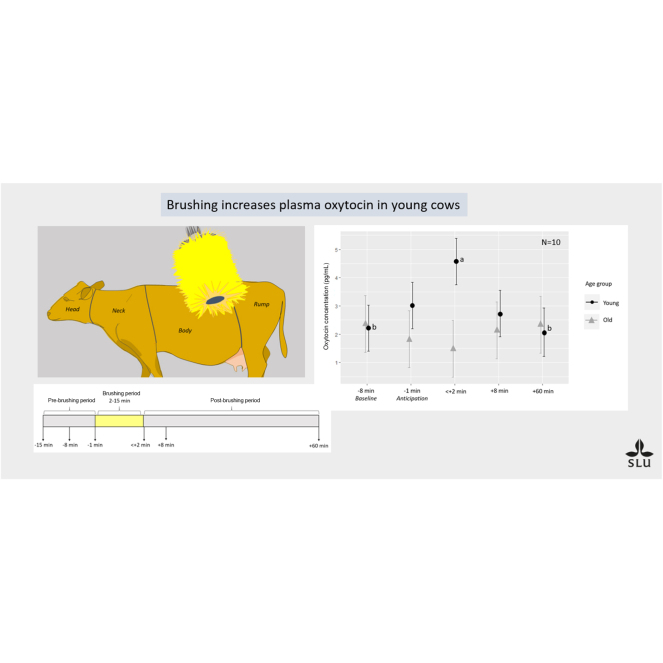
**Summary:** The aim of this study was to investigate whether plasma oxytocin (OT) in cows is affected when they groom themselves using a mechanical rotating brush and whether the brushed body region influences OT response. We found that plasma OT levels were significantly higher within 2 minutes following brush use compared with pre-brushing levels, but this effect was observed only in the 6 younger cows, which had experienced a maximum of 2 lactation periods. We also found that longer durations of brushing on the core body region, a type of brushing that was more pronounced among older cows, correlated with decreases in OT levels.

It is currently common practice to equip loose housing systems for dairy cattle with mechanical brushes, with suggested welfare benefits through promoting comfort behaviors (EFSA AHAW Panel, 2023). Cows have high motivation to use the brushes (McConnachie et al., 2018) and use them frequently following installation (∼7.71 visits/d in groups of 12 cows observed in DeVries et al., 2007), with later average daily durations of ∼108 to 357 s/d (Mandel and Nicol, 2017). Although the direct physiological effects of mechanical brush use are unknown, increased brush use has been observed during the first hour after milking (Mandel and Nicol, 2017), and higher brush use has been connected to greater milk yield (Keeling et al., 2016). This suggests a potential link to the oxytocin (**OT**) release chain, as OT levels rise during successful milking (Bruckmaier et al., 1994). Investigating the direct physiological responses to brushing is essential for understanding the role of brushes for positive emotional states (Arndt et al., 2022), and OT remains a relevant measure to investigate in this context (Rault et al., 2017).

A neuropeptide, OT is produced in the hypothalamus and stored in the posterior pituitary until being released into the blood. Basal values of OT concentration in blood are extremely low, around <5 pg/mL when investigated in lactating and nonlactating (dry) beef cattle (Wagner et al., 2021) and much lower than most hormones derived from peripheral endocrine glands. Playing a role in reproduction, OT is released into peripheral circulation through a neuro-endocrine reflex, primarily triggered by tactile stimulation of the teats or genital tract (Bruckmaier et al., 1994). In addition to being released into blood, OT is also secreted in the central nervous system, where it is believed to facilitate and maintain a strong bond between mother and offspring (Nagasawa et al., 2012) and modulate other social behaviors (Caldwell, 2017). The adaptive value of social bonds is thought to have facilitated an experience of positive emotions associated with OT release during skin-to-skin contact (Spruijt et al., 1992). Mechanical brush access has been seen to increase maternal behavior in cows (Newby et al., 2013), suggesting that brush use may enhance OT exposure (Champagne et al., 2001). Nevertheless, relatively little information is available in cows about the effects of OT beyond milk ejection or about OT release in response to stimulation to other parts of the body than the udder or genitals.

That manual brushing can increase OT levels in calves (Miranda et al., 2023) supports the possibility of calming effects and pleasant feelings from low-intensity tactile stimulation in ruminants similar to those previously found in other mammals (Uvnäs-Moberg et al., 2015). However, another study found no similar effect of manual brushing among older cows during milking (Wredle et al., 2022), suggesting that either the maximum OT threshold was already reached during milking or there were no OT effects of brushing in the particular way it was performed in that study (i.e., controlled by the human and in a tiestall).

The aim of this study was to investigate whether plasma OT is affected in cows when they groom themselves using a stationary mechanical rotating brush, where they have control over how and where on the body they brush. We predicted that this self-grooming would lead to increased release of OT into the blood. Such a finding could support the view that brush use is associated with positive emotions and that brush use can be a potential positive welfare indicator, as suggested by Keeling et al., (2021). In addition, we wanted to explore whether brushing on specific body regions could influence the OT increase, as heart rate and behavioral differences have been found following allogrooming (Laister et al., 2011) and human stroking (Schmied et al., 2008a,b) of different cow body regions.

This experiment was approved by the Animal Research Ethics Committee in Uppsala, Sweden (Protocol number C 58/13) and conducted at the Swedish Livestock Research Centre at the Swedish University of Agricultural Sciences in Uppsala, Sweden. The test subjects and experimental units were 12 dry dairy cows of the Swedish Red breed, housed and handled according to standardized procedures at a commercial farm before the experiment. Dry cows were selected to eliminate any potential effects of time since last milk ejection on OT levels in the blood. Inclusion criteria were that the cow had previous experience of the mechanical brush (brush access was provided during their lactation period), were social toward humans by being easy to handle (as the test procedure involved moving cows using a halter) and had an expected calving date that allowed the tests to be completed at least 2 wk before expected parturition. The interval between the test session and calving turned out to be on average 20 d (SD ± 8, range 10–33 d). Each cow was brought to the experimental area on a rolling schedule (maximum of 4 cows at any time), at least 2 wk before the first test session for habituation to the environment, test equipment, and researchers. The experimental area was located in the same building as their previous home pen and consisted of a holding pen, a test area, and a brush area (Figure 1a). The cows were kept continuously in the holding pen, which contained 5 cubicles, feeding stations, and a drinker. The cows had access to the test area and the brush area only during training sessions (when they were being trained to enter the brush area to access the brush), or during a test session. All cows were in the test and brush area at least 5 times on 5 different days before being tested, and all were seen using the brush (DeLaval swinging cow brush SCB3) during these times. Cows were tested only once. As OT levels can vary among individuals, each cow served as its own control (i.e., levels before vs. after brushing were compared). The area in the holding pen closest to the brush was closed off during testing to prevent any physical contact between cows in the holding pen and the test cow, but they could always see and hear each other. The feeding stations in the test area were blocked during the test session, but water was always available ad libitum.

**Figure 1:**
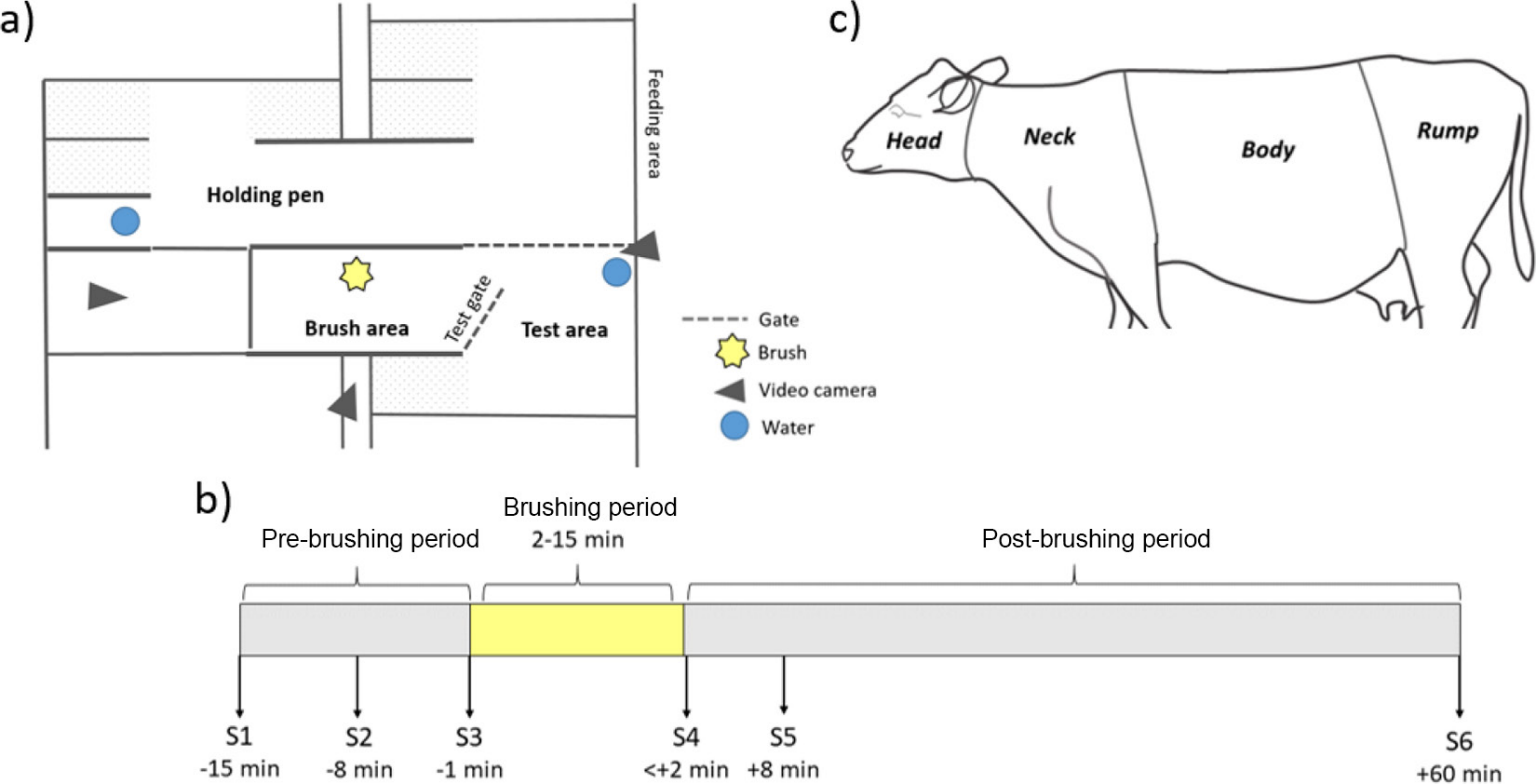
Overview of the methodology, with (a) an overview of the experimental area; (b) an overview of the test sessions and the timing for each blood sample (S), where the length of the brushing period varied depending on the duration of the cow's own brushing behavior; and (c) the body regions used when observing brushing duration on specific parts of the body.

Each test session involved a catheterization procedure, a recovery period, a pre-brushing period, a brushing period, and a post-brushing period (Figure 1b). During catheterization, a cow was let into the test area and a semipermanent catheter was placed in the jugular vein in the lower neck region. To limit movement, the cow's head was inside the self-locking stanchions. The area around the jugular vein was shaved and cleaned with alcohol-soaked cotton balls, and a subcutaneous injection of 1 mL of procaine (Procamidor Vet, 20 mg/mL, Salfarm Scandinavia) was given. A semipermanent catheter (Mila 2.1 × 90 mm, Swevet) was inserted, and secured with 2 sutures (Supramid USP 3, Braun). An elongation tube (Discofix 20 cm, Braun) with a 3-way opening was added and attached with tissue glue (Vetbond, 3M). The loose end was either protected with an Animal Polster dressing (Animal Polster, Snögg) or taped to a heart rate belt. The catheter and extension tube was flushed with 10 mL (2× the volumes) of heparinized (10 IU of heparin/mL made from Heparin LEO 5000 IU/mL, Leo Pharma) saline to avoid clotting between samples. A recovery period of 2 h with access to feed and water before the 15-min pre-brushing period was based on the time taken for heart rate and behavior to return to baseline in a pilot study.

After the pre-brushing period, the gate to the brush area was opened, allowing the cow to enter voluntarily. All cows entered and began brushing within 2.9 s on average (SD ± 2.8). Brushing was defined as body contact with a rotating brush or active body movements (e.g., rubbing) against a nonrotating brush for at least 2 s, and a pause was defined as no body-brush contact or no rubbing against a nonrotating brush for at least 2 s. Total brushing duration was determined using these criteria, similar to Mandel et al. (2013). Brushed body regions (head, neck, body, rump; Figure 1c) were recorded instantaneously every 5 s and were not mutually exclusive. The brushing period varied, as we did not want to disturb the brushing, and ended either when a cow paused brushing for 15 s after at least 2 min of brushing, or brushed continuously for 15 min. The 2-min threshold was based on the lag time between teat stimulation and onset of milk ejection (Bruckmaier et al., 1994). When the brushing period ended, the brush was powered off, and a 60-min post-brushing period followed. The same person placed all catheters and collected blood samples. Pre-brushing samples (**S1**–**S3**) were taken 15, 8, and 1 min before gate opening (Figure 1b). Sample S1 was excluded due to possible influence from the return of the experimenters after the solitary recovery period (similar to Rehn et al., 2014). Sample S2 served as pre-brushing baseline, and S3 was considered to potentially include anticipation effects. The first 2 post-brushing samples (**S4** and **S5**) were taken in the brush area to avoid any influence of the movement back to the test area: S4 within 2 min after brushing (mean 88 ± SD 28 s) to assess immediate effects, and S5 and S6 at 8 and 60 min to track OT levels' return to baseline. The sample period included balanced time points before and after brush use.

During blood sampling, cows were loosely held by a lead rope attached to a halter, which provided safety and efficiency. It was part of the selection process of the cows that they accepted to be held in this way. For S2, S3, and S5, the cows were already being held, whereas for other samples, an experimenter calmly attached the lead rope.

At each blood sampling, the catheter content was discarded and blood was drawn into 4.9-mL Na-EDTA Monovette tubes (Sarstedt). The catheter tube was then refilled with anticoagulant. The whole procedure took around 2 min. Samples were placed on ice within 2 min and centrifuged within 50 min (1,500 × *g* relative centrifugal force, 4°C, 20 min) and then stored at −20°C. Plasma OT concentration was determined via radioimmunoassay after extraction with Sep Pak C18 cartridges (Waters, Dublin, Ireland) as described by Schams (1983). Samples were coded and analyzed by blinded researchers.

Cows had gone through at least 1 lactation period but varied in their number of finished lactation periods. To investigate or control for these differences, as OT release may be age-dependent (Elabd et al., 2014), cows were divided into 2 age groups (young n = 7; old n = 5) around the average number of finished lactation periods (mean ± SD: 2.6 ± 1.2). Two cows, one from each age group, were excluded from the analysis. One of these cows did not use the brush for the minimum required brushing duration of 2 min, and the other was excluded due to problems during the catheterization. Furthermore, there were problems collecting blood at the last sample point (S6) for one young cow. It is possible that the cow not using the brush experienced the catheterization procedure as too aversive. Cows have been seen to use a mechanical brush less if it is located farther away from the feed as well as during periods of discomfort, such as during high temperature or humidity (Mandel et al., 2013) and during social stressors (Lecorps et al., 2020).

All statistical analyses were conducted using R Studio (version 3.6.1, R Core Team). Graphs were plotted in R package ggplot2 (Wickham, 2016). The OT levels at different samples points were investigated using linear mixed models fitted by REML in R package lme4 and Satterthwaites's method (Bates et al., 2015). The model included individual as a random effect and cow age (young or older) in a fixed interaction effect along with timing of the sample (S2–S6). Model assumptions were checked by diagnostic plots using the DHARMa-package (Hartig, 2022). Fixed effects, including their interactions, were investigated in Type III Wald *F* tests with Kenward-Roger approximation. Significant main effects (*P* < 0.05) and interactions showing a trend (*P* < 0.1) were investigated further in pairwise comparisons in the emmeans-package (Lenth, 2022) with Kenward-Roger approximation and Tukey method for multiple comparison. The relationships between the differences in OT levels before (S2) and after brushing (S4) were compared with total brushing duration, as well as with the duration of brushing specific regions (head, neck, body, rump) using a nonparametric Spearman rank correlation test. The effect of age on duration of brushing specific regions was investigated using the Kruskal-Wallis rank sum test.

During the brushing period, cows brushed on average 92% (SD ± 7) of the time. That it was easy to train them to enter the brush area, and that almost all started brushing immediately following brush access, supports earlier work that cows are motivated to use a mechanical brush. For example, cows have been seen to work as hard to access a mechanical brush as to access fresh feed (McConnachie et al., 2018). The cows had an average brushing duration of 412 s (SD ± 279). The head was brushed on average for 31 s (SD ± 63; 7%), the neck for 57 s (SD ± 81; 14%), the body for 94 s (SD ± 145; 23%), and the rump for 232 s (SD ± 246; 56%).

The total brushing duration was not lower than that seen in another study with a similar test setup but without additional equipment on the cow (Lecorps et al., 2021), and was even found to be longer than those observed for individuals in a milking herd where cows shared brush access (Mandel and Nicol, 2017; Foris et al., 2023). As a first conclusion, the test procedure did not seem to reduce cows brush use. However, it could have altered how the cows used the brush. Lower proportions of head brushing were indeed seen when compared with 2 other studies (DeVries et al., 2007; Burton and Blackie, 2024). However, this comparison could also be explained by brush differences, as nonrotating and nonswinging brush types may lead to more active brushing, often involving the head. The proportions of brushed body regions were similar to those found in a study with same brush type, among lactating cows of the same breed in the same herd when in their home area (de Oliveira et al., 2015).

Plasma OT levels were found to be influenced by the interaction of sample point and age (*F*_4,31_ = 2.63, n = 10, *P* = 0.053). Pairwise comparisons of sample differences, stratified on age, showed that OT levels in the first blood sample after brushing (S4: 4.57 ± 0.82 pg/mL), among younger cows, was significantly higher compared with the baseline sample (S2: 2.21 ± 0.82 pg/mL; n = 6, *P* = 0.026) and the sample 60 min after brushing (S6: 2.07 ± 0.82 pg/mL; n = 5, *P* = 0.026; Figure 2). The proportional OT increase observed in younger cows in this study is similar to the response seen in calves after 4 min of manual brushing by a human (Miranda et al., 2023), although it remains lower than that seen during milking (Wredle et al., 2022). Importantly, the effect of OT on milk ejection is not solely dependent on the magnitude of its release. It has been demonstrated that even a slight increase in OT concentration, by just a few picograms per milliliter above baseline, can trigger milk ejection in dairy cows (Bruckmaier et al., 1994; Watters et al., 2015), indicating that even small fluctuations in OT levels can have important physiological effects. Furthermore, potential negative experiences of the catheterization procedure, pregnancy, and limited social contact may have influenced the brushing as well as the OT response.

**Figure 2:**
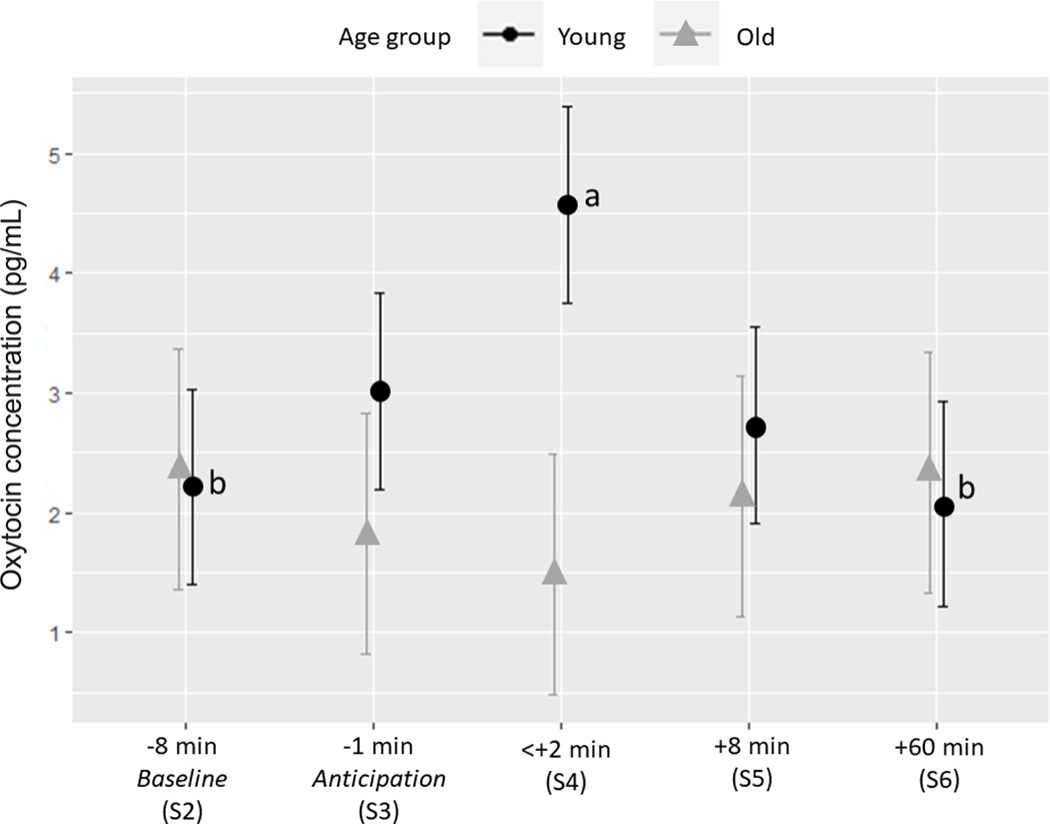
Oxytocin (OT) concentrations (mean ± SE) around brushing were influenced by cow's age, where OT increase after brushing was found to be significant only for younger cows (maximum 2 previous lactation periods). Different letters (a,b) indicate significant differences between sample points within age groups.

No sample differences were found among the samples in older cows (*P* ≥ 0.05; Figure 2), which may be explained by a lower physiological response to brushing or by differences in the OT release mechanism. Prior research indicates that plasma OT levels generally decrease with age (Elabd et al., 2014), but baseline levels in our study did not differ among cows of varying age. It is possible that an OT peak in older cows could have occurred earlier or declined more rapidly. Oxytocin has a short plasma half-life and decreases exponentially following an increase in concentration (Belo and Bruckmaier, 2010), so we could have missed the potential OT peak, as sampling was not possible during brushing. Mandel and Nicol (2017) found that younger cows had a more pronounced increase in brush use postpartum and interpreted this as a redirected maternal behavior, supporting the view of physiological differences around brush use among cows of varying age.

Another possibility is that cows of varying ages used the brush differently. Other studies have observed that age can influence cows' brushing behavior regarding frequency and duration (Keeling et al., 2016; Mandel and Nicol, 2017). We found no differences between the 2 age groups in overall brushing duration or time spent brushing head, neck, or rump (n = 10, *P* > 0.05), but older cows were found to spend longer time brushing the core body (young: 32.4 ± 26.5 s, old: 186.57 ± 206.29 s; χ^2^ = 6.55, n = 10, *P* = 0.01).

Increased OT following brushing (S4–S2) had a positive correlation with duration of brushing the head and neck regions (head: rho = 0.26, n = 10, *P* = 0.46; neck: rho = 0.10, n = 10, *P* = 0.78), whereas a negative correlation was found for total, body, and rump brushing duration (total: rho = −0.41, n = 10, *P* = 0.25; body: rho = −0.927, n = 10, *P* < 0.001; rump: rho = −0.35, n = 10, *P* = 0.33). Only the negative relationship between OT increase and brushing duration of the core body region was strong and significant.

That brushing on different body regions could lead to differences in OT release is supported by previous studies investigating allogrooming (Laister et al., 2011) and human stroking (Schmied et al., 2008a,b) and their effects on behavior and heart rate. Specifically, allogrooming or stroking of the neck region, including the withers, seems to lead to greater relaxation in cows, based on reduction in heart rate and greater prevalence of neck stretching and ear hanging. This could be explained by neurophysiological differences at different regions, leading to differences in the sensation experienced by the cow when being touched or brushed, but it could also be that certain body regions are associated with memory of a sensation, such as during social bonding (Nagasawa et al., 2012; Caldwell, 2017). Even if the relationships between brushing duration of different body regions and changes in OT levels were weak, and in most cases nonsignificant, the findings are worthy of further investigation. For example, one might expect a quadratic relationship between brushing duration and OT levels following brushing, as OT release could require a minimum time of brushing stimulation, whereas a longer brushing duration could risk going beyond the OT peak level, and so an OT increase after brushing could be undetected. However, due to the low number of animals, we could not perform this type of analysis.

Another possible explanation for the age-related differences in OT increase could be brushing intensity. Older cows may have been more passive, resulting in longer core body brushing, whereas younger cows may have shifted brushed regions more frequently, indicating greater arousal. Because arousal can influence OT release (Rault et al., 2017), brushing intensity and its effects merit further study.

No evidence of an anticipation effect in OT levels was found in the sample just before the brush access (S3), as S2 and S3 were not significantly different (*P* ≥ 0.05; Figure 2).

Although we used each cow as its own control, we acknowledge that the limited sample size warrants caution in interpretation of the results. Repeating the study, particularly with nonpregnant and lactating cows, could strengthen the findings. Although selecting socially inclined cows may have influenced the results, balancing sociality would require a much larger sample size and could complicate the sampling procedure.

In conclusion, mechanical brush use increased OT levels in younger cows. Although the increase in plasma OT concentration was small, it may hold biological relevance. Potential OT increase during brushing in older cows remains to be confirmed in further studies. All cows voluntarily used the brush, implying that they experienced it as rewarding. Our results support that brushing can be linked to positive emotions, at least in younger cows. Brushed body region seemed to influence the OT changes, warranting further studies, particularly when exploring the potential of using brushing behavior as a positive welfare indicator.
